# Genetic Defects and Pro-inflammatory Cytokines in Parkinson's Disease

**DOI:** 10.3389/fneur.2021.636139

**Published:** 2021-06-22

**Authors:** Albert Frank Magnusen, Shelby Loraine Hatton, Reena Rani, Manoj Kumar Pandey

**Affiliations:** ^1^Division of Human Genetics, Cincinnati Children's Hospital Medical Center, Cincinnati, OH, United States; ^2^Department of Paediatrics of University of Cincinnati College of Medicine, Cincinnati, OH, United States

**Keywords:** neuroimmunology, immunogenetics, innate and adaptive immunity, glycosphingolipid, aggregated proteins, brain disease, neuroinflammation, mitochondrial disease

## Abstract

Parkinson's disease (PD) is a movement disorder attributed to the loss of dopaminergic (DA) neurons mainly in the substantia nigra pars compacta. Motor symptoms include resting tremor, rigidity, and bradykinesias, while non-motor symptoms include autonomic dysfunction, anxiety, and sleeping problems. Genetic mutations in a number of genes (e.g., *LRRK2, GBA, SNCA, PARK2, PARK6*, and *PARK7*) and the resultant abnormal activation of microglial cells are assumed to be the main reasons for the loss of DA neurons in PD with genetic causes. Additionally, immune cell infiltration and their participation in major histocompatibility complex I (MHCI) and/or MHCII-mediated processing and presentation of cytosolic or mitochondrial antigens activate the microglial cells and cause the massive generation of pro-inflammatory cytokines and chemokines, which are all critical for the propagation of brain inflammation and the neurodegeneration in PD with genetic and idiopathic causes. Despite knowing the involvement of several of such immune devices that trigger neuroinflammation and neurodegeneration in PD, the exact disease mechanism or the innovative biomarker that could detect disease severity in PD linked to *LRRK2, GBA, SNCA, PARK2, PARK6*, and *PARK7* defects is largely unknown. The current review has explored data from genetics, immunology, and *in vivo* and *ex vivo* functional studies that demonstrate that certain genetic defects might contribute to microglial cell activation and massive generation of a number of pro-inflammatory cytokines and chemokines, which ultimately drive the brain inflammation and lead to neurodegeneration in PD. Understanding the detailed involvement of a variety of immune mediators, their source, and the target could provide a better understanding of the disease process. This information might be helpful in clinical diagnosis, monitoring of disease progression, and early identification of affected individuals.

## Introduction

Parkinson's disease (PD) is a neurodegenerative brain disorder that mainly happens due to progressive loss of dopaminergic (DA) neurons in the substantia nigra pars compacta (SNPC) and its impact on impairment of motor function that includes static tremor, bradykinesia, muscle stiffness, postural instability, balance difficulty, and walking problem (1, 2). Pro-inflammatory cytokines and chemokines have been linked to disease manifestations of Alzheimer's disease, multiple sclerosis, Huntington's disease, amyotrophic lateral sclerosis, prion disease, systemic lupus erythematosus, depression, migraine, and schizophrenia as reviewed in refs. (3–12). Microglial cells (MGCs) are residential macrophages (Mϕs) of the central nervous system (CNS), which are exquisitely sensitive to the pathophysiological insults and the resultant alteration in their morphology and phenotype to activated state (13). Such MGCs cause massive generation of pro-inflammatory cytokines, chemokines, reactive oxygen species (ROS), and nitric oxide (NO), which all contribute to the clearance of infectious agents (14). However, prolonged or excessive activation of MGCs results in pathological forms of inflammation that contribute to the progression of neurodegenerative and neoplastic diseases (15–17). Activated MGCs express major histocompatibility complex II (MHC class II), which is required for activation of naive CD4^+^ T cells and the production of numerous pro-inflammatory cytokines and chemokines that modulate the differentiation of effector T cells (18).

Effector T cells, i.e., T helper 1 (Th1), Th2, Th17, T regulatory (Treg), and T follicular helper (Tfh) cells as well as their signature cytokines, i.e., interferon gamma (IFNγ; TH1), interleukin 4 (IL-4; TH2) (19, 20), IL-17 (TH17) (21, 22), transforming growth factor beta (TGFβ; Treg), and IL-6 (Tfh), drive tissue inflammation in several visceral and brain diseases (23–28). The T helper cell subsets can produce IL-10, a cytokine with broad immunoregulatory properties (29). Th1 cells produce IFNγ, IL-2, and tumor necrosis factor alpha (TNFα) to clear intracellular pathogens and evoke cell-mediated immunity, whereas Th2 cells produce IL-4, IL-5, and IL-13 to clear extracellular organisms and evoke strong allergic responses (19, 30–33). In contrast to Th1 and Th2 cell differentiation, which depend on their respective effector cytokines (IFNγ and IL-4), Th17 cell differentiation does not require IL-17 but has a critical need for TGFβ and IL-6 (34–36). Treg cells produce IL-10 and TGFβ to cause immune tolerance and inhibit IFNγ synthesis (37) as well as block T helper cell differentiation of naive T cells into effector T cells (38).

The MGCs' interaction to effector T cells and the resulting production of pro-inflammatory cytokines, chemokines, and the neurodegeneration have been observed in Alzheimer's disease, amyotrophic lateral sclerosis, multiple sclerosis (MS), and prion diseases (17, 39, 40). The SNPC of PD patients have shown CD4^+^ T cells, CD8^+^ T cells, human leukocyte antigen DR isotype (HLA-DR) expressing inflammatory subset of MGCs, and increased incidence of pro-inflammatory cytokines, i.e., IFNγ, TNF, IL-1β expressing glial cells (41–43). Additionally, the striatal dopaminergic (DA) regions and cerebrospinal fluid (CSF) of PD patients have shown elevated levels of IL-1β, IL-2, IL-6, TNF, and TGFβ1 (44, 45). Peripheral blood analyses of PD patients have shown marked increases of innate and adaptive immune cells that include monocytes (MOs), IFNγ, IL-4, and IL-17 producing memory and effector T cells as well as their association to severity of the disease (43, 46–51). Elevated serum levels of TNF (52, 53), IL-1β (52, 54, 55), and IL-6 (52–54) have been observed in PD patients as reviewed in Qin et al. (56). PD patients have also shown increased serum level of cytokine receptors such as TNF receptors (e.g., TNFRs) and their link to late disease onset (57, 58). MO differentiation into the tissue-specific MGCs, Mϕs, and dendritic cells (DCs) as well as the trafficking of CD4^+^ and CD8^+^ T cells to sites of inflammation requires growth factors, i.e., granulocyte colony-stimulating factor (GCSF), granulocyte Mϕ colony-stimulating factor (GMCSF), and the Mϕ colony-stimulating factor (MCSF), as well as the number of C-C motif ligand (CCL) and the C-X-C motif ligand (CXCL) chemokines (59–69). However, the exact mechanism by which such immune inflammation occurs in PD is unknown. It is speculated that abnormal brain or circulatory level of several proteins and enzymes has been associated with the development of neuroinflammation in PD. Indeed, several of such proteins have been associated with activation of residential MGCs and the infiltrated lymphocytes and their combined impact on the generation of pro-inflammatory cytokines (e.g., IFNβ, IFNγ, TNFα, IL-1β, IL-6, IL-18, and TGFβ1), which lead to the loss of DA neurons in 1-methyl-4-phenyl-1,2,3,6-tetrahydropyridine (MPTP) or 2,4,5-trihydroxyphenethylamine or 6-hydroxydopamin (6-OHDA)-induced mouse models of idiopathic PD (Table 1A). Additionally, human patients with idiopathic PD have also suggested elevated brain or circulatory level of proteins or enzymes linked to MGC activation, pro-inflammatory cytokine and chemokine (e.g., IFNβ, IFNγ, TNFα, IL-1β, IL-2, IL-4, IL-6, IL-10, IL-12, IL-13, CCL2, CXCL1) production, loss of DA neurons, and the development of motor symptoms (Table 1B). The current review is an update on the involvement of a variety of innate and adaptive immune mediators as well as their source and targets involved in the propagation of disease manifestations in mouse and human PD associated with *LRRK2, GBA, SNCA, PARK2, PARK6*, and *PARK7* defects. These results will likely provide much needed insights into the disease mechanism and will be useful for the identification of potential biomarkers at the level of distinguished cytokines and chemokines in different forms of PD.

**Table 1A:** Cytokines and their source in the mouse model of idiopathic PD.

**PD mouse model**	**Proteins/enzymes and their source**	**Pro-inflammatory cytokines, chemokines, and their source**		**Brain defects**	**References**
MPTP- and 6-OHDA-induced disease	Striatum^α−syn (P+)^ Striatum^TH (P−)^ Striatum^DA (P−)^ SNPC^α−syn (P+)^ SNPR^α−syn (P+)^ Thalamus^α−syn (P+)^ DG^α−syn (P+)^ AON^α−syn (P+)^ OB^α−syn (P+)^ MC^α−syn (P+)^ SC^α−syn (P+)^ OC^α−syn (P+)^	IFNγ	SNPC^P+^ Sera^P+^ Striatum^P+^	DA neuron death in SN, striatum, and NP	(70–72)
MPTP- and R-APO-induced disease	Striatum^TH (P−)^ NP^NOSP (P+)^ NP^OSPA170 (P+)^ MGCs^NADPHoxidase (P+)^ ACs^Oxidase (P+)^ MGCs^iNOS (P+)^	TNFα	SNPC^P+andM+^ Sera^P+andM+^ Striatum^P+^ CP^M+^	DA neuron death in SN, striatum, and NP	(71–76)
MPTP-induced disease	Striatum^TH (P−)^ NP^NOS (P+)^ NP^OSPA170 (P+)^ MGCs^NLRP3 (P+)^ MGCs^NADPHoxidase (P+)^ ACs^myeloperoxidase (P+)^ ACs°*xidase* (*P*+) MGCs^iNOS (P+)^ SN-MGCs^NLRP3 (P+)^ SN-ACs^NLRP3 (P+)^ NCs^Caspase1 (P+)^	IL-1β	SNPC^P+^ Sera^P+^ Striatum^P+^ SN^M+^ CP^M+^ MB^M+^	MGC activation, DA neuron death in SN, striatum, and NP	(71–78)
MPTP- and R-APO-induced disease	Striatum^TH (P−)^ NP^NOS (P+)^ NP^OSPA170 (P+)^	IL-6	CP^M+^	DA neuron death in SN, striatum, and NP	(71–74)
MPTP-induced disease	SN-MGCs^NLRP3 (P+)^ SN-ACs^NLRP3 (P+)^ NCs^Caspase1 (P+)^	IL-18	MB^M+^	MGC activation, DA neuron death in NP	(78)
MPTP-induced disease	Striatum^TH (P−)^	TGFβ1	Striatum^P+^	DA neuron death in NP	(72)

*NCs, neurons; NP, nigrostriatal pathway; ACs, astrocytes; MGCs, microglial cells; α-syn, alpha-synuclein; TH, tyrosine hydroxylase; DA, dopamine; SN, substantia nigra; SNPC, substantia nigra pars compacta; SNPC, substantia nigra pars compacta; SNPR, substantia nigra par reticulata; DG, dentate gyrus; Hipp, hippocampus; AON, anterior olfactory nucleus; OB, olfactory bulb; MC, motor cortex; SC, sensory cortex; OC, orbital cortex; CP, caudate putamen; IFN, interferon; TNF, tumor necrosis factor; TGF, transforming growth factor; IL, interleukin; α, alpha; β, beta; γ, gamma; MPTP, 1-methyl-4-phenyl-1,2,3,6-tetrahydropyridine; 6-OHDA, 6-hydroxydopamine; R-APO, R-apomorphine; NOS, nitric oxide synthase; OSPA170, oxidative stress-induced Protein A 170; M, mRNA expression; P, protein expression; +, increased level; –, decreased level; **∞**, no change; ND, no data*.

**Table 1B:** Cytokines and their source in idiopathic human PD.

**Human PD**	**Proteins/enzymes and their source**	**Pro-inflammatory cytokines and chemokines and their source**		**Brain defects**	**References**
Idiopathic	Sera^PINK1 (M/P−)^ Sera^Parkin^ ^(M/P−)^	IFN-β1	Sera^P+^	Inflammation, motor defects, and loss of DA neurons	(79)
Idiopathic	Sera^TBARS (P+)^ Lymphocytes ^(P+)^	IFN-γ	Sera^P+^	Damage of DA neurons in nigrostriatal regions	(71)
Idiopathic	Sera^TBARS (P+)^ Lymphocytes ^(P+)^ Fibroblast^COX−2 (M+)^	TNFα	Sera^P+^ Fibroblast^P+^ Blood^P+^	Inflammation, damage of DA neurons in nigrostriatal regions	(56, 71, 80)
Idiopathic	Sera^TBARS (P+)^ Lymphocytes ^(P+)^	IL-1β	Sera^P+^ Blood^P+^	Inflammation, damage of DA neurons in nigrostriatal regions	(56, 71, 81)
Idiopathic	Sera^TBARS (P+)^ Lymphocytes ^(P+)^	IL-2	Sera^P+^	Inflammation, damage of DA neurons in nigrostriatal regions	(71, 73, 82)
Idiopathic	Sera^TBARS (P+)^ Lymphocytes ^(P+)^	IL-4	Sera^P+^ Blood^P+^	Inflammation, damage of DA neurons in nigrostriatal regions	(56, 71)
Idiopathic	Sera^TBARS (P+)^ Lymphocytes ^(P+)^ Fibroblast^COX−2 (M+)^	IL-6	Sera^P+^ Fibroblast^P+^ Blood^P+^ Plasma^P+^	Inflammation, damage of DA neurons in nigrostriatal regions	(56, 71, 80, 83)
Idiopathic	CRP ^(P+)^	IL-10	Blood^P+^	Inflammation, damage of DA neurons in nigrostriatal regions	(56)
Idiopathic	Sera^PINK1 (M/P−)^ Sera^Parkin (M/P−)^	IL-12	Sera^P+^	Inflammation, motor defects, and loss of DA neurons	(79)
Idiopathic	Sera^PINK1 (M/P−)^ Sera^Parkin^ ^(M/P)^	IL-13	Sera^P+^	Inflammation, motor defects, and loss of DA neurons	(79)
Idiopathic	Sera^PINK1 (M/P−)^ Sera^Parkin^ ^(M/P−)^	CCL2/MCP1	Sera^P+^	Inflammation, motor defects, and loss of DA neurons	(79)
Idiopathic	Sera^PINK1 (M/P−)^ Sera^Parkin (M/P−)^	CXCL1/KC	Sera^P+^	Inflammation, motor defects, and loss of DA neurons	(79)
Idiopathic	Blood^CRP (P+)^ CSF^CRP (P+)^	hs-CRP	Sera^P+^ Blood^P+^ Plasma^P+^ CSF^P+^	Inflammation, loss of DA neurons	(56, 84)

*PINK1, PTEN-induced kinase 1; Parkin, 465-residue E3 ubiquitin ligase; TBARS, thiobarbituric acid reactive substances; IFN, interferon; TNF, tumor necrosis factor; TGF, transforming growth factor; IL, interleukin; α, alpha; β, beta; γ, gamma; CXCL1, chemokine C-X-C motif ligand-1; CCL2, chemokine C-C motif ligand-2; CRP, C-reactive protein; hs-CRP, high sensitivity C-reactive protein; CSF, cerebrospinal fluid; COX-2, cyclooxygenase-2; DA, dopaminergic; M, mRNA expression; P, protein expression; +, higher/increased levels; –, decreased/lower levels; **∞**, no change; ND, no data*.

## *LRRK2* Gene Defects and Pro-inflammatory Immune Mediators in PD

The leucine-rich-repeat kinase 2 (*LRRK2*) gene encodes a large, multidomain LRRK2 protein comprised of a GTPase and a kinase domain (85). Although the precise physiological function of LRRK2 remains largely unknown, recent studies have indicated that LRRK2 is involved in cellular functions such as neurite outgrowth, cytoskeletal maintenance, vesicle trafficking, autophagic protein degradation, and the regulation of signaling pathways, including the Wingless-INT (WNT), Fas-Fas ligand (FasL or CD95L or CD178)-associated protein with death domain (FADD), mitogen-activated protein kinase (MAPK), and nuclear factor κ-light-chain-enhancer of activated B cells (NF-κB) (86–88).

The resting neuronal cells, i.e., neurons (NCs), MGCs, and astrocytes (ACs), expressed a low level of LRRK2 (89, 90). However, several of the pro-inflammatory mediators (e.g., IFNβ, IFNγ, TNFα, IL-6, and LPS) cause upregulation of LRRK2 in immune cells, i.e., monocytes (MOs), Mϕs, and T and B cells, and in neuronal cells, i.e., MGCs and NCs (88, 91–95). LRRK2 is critical for the propagation of Crohn's disease (96, 97), leprosy (98), and neuronal toxicity (99–102).

**Figure 1:**
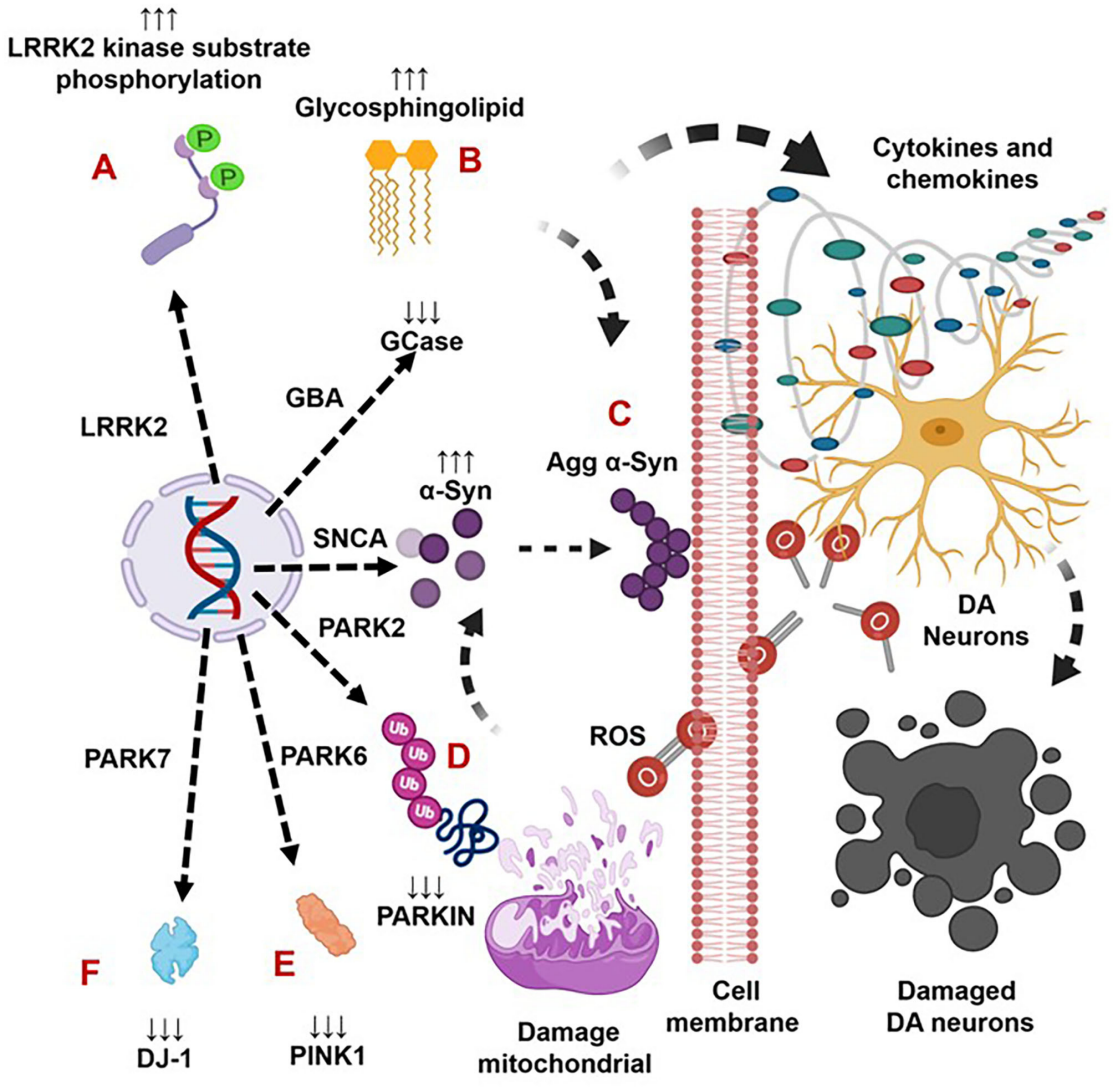
The genetic mutation-induced inflammatory immune reactions develop neurodegeneration in Parkinson's disease. The LRRK2 defects cause over activation of LRRK2 kinases. This defect triggers the formation of aggregated alpha-synuclein (Agg α-syn) and increased generation of pro-inflammatory cytokines and chemokines that lead to the loss of dopaminergic (DA) neurons in LRRK2-associated PD **(A)**. The *GBA* mutations and the resultant deficiency of glucocerebrosidase (GCase) trigger the formation of glycosphingolipids and Agg α-syn, which trigger increased generation of pro-inflammatory cytokines and chemokines and lead to the loss of DA neurons in GBA-associated PD **(B)**. The SNCA defects and the resultant overproduction of normal/Agg α-syn activate the brain production of inflammatory cytokines and chemokines that cause death of DA neurons in SNCA-associated PD **(C)**. The PARK2, PARK6, and PARK7 defects and the subsequent deficiency of PARKIN, PINK, and DJ-1 proteins cause mitochondrial damage and the formation of Agg α-syn. These abnormalities trigger cellular activation and massive generation of ROS, pro-inflammatory cytokines, and chemokines that lead to the loss of DA neurons in PARK2-, PARK6-, and PARK7-associated PD **(D–F)**.

Indeed, *LRRK2* gene mutations have been linked to increased LRRK2 kinase substrate phosphorylation and the formation of intracellular alpha-synuclein (α-syn)-positive inclusions in Lewy bodies (LBs) and preferential loss of DA neurons and the development of motor symptoms, including tremor, rigidity, postural instability, and bradykinesia in late-onset familial and idiopathic PD (100, 103–119). The brain regions, blood, and cells of LRRK2-associated mouse models of PD have shown abnormal expression of LRRK2 kinase and their association with elevated brain and circulatory level of pro-inflammatory cytokines (e.g., IFNγ, TNFα, IL-1α, IL-1β, IL-6, IL-8, IL-10, and IL-12), chemokines (e.g., CCL2, CCL3, CCL4, CCL5, CXCL1, and CXCL10), and growth factors (e.g., GCSF and MCSF), as well as their link to the loss of NCs and the development of cognitive defects (Table 2A). The blood cells, sera, and CSF of LRRK2-associated human patients with PD have also shown abnormal expression of LRRK2 kinase and their link to elevated levels of pro-inflammatory cytokines and growth factors (e.g., IFNγ, TNFα, IL-1β, IL-2, IL-4, IL-6, IL-8, IL-10, IL-12, IL-13, GCSF, PDGF, and VEGF), loss of NCs, and the development of cognitive defects in PD (Table 2B). These data suggest that LRKK2 defects and the resultant higher expression of LRRK2 kinases cause cellular activation and the higher generation of pro-inflammatory cytokines and chemokines (Tables 2A,B) that lead to DA neuron damage in LRRK2-associated PD (Figure 1A).

**Table 2A:** Cytokines and their source in the LRRK2 mouse model of PD.

**PD mouse model**	**LRRK2 kinase level and its source**	**Pro-inflammatory cytokines, chemokines, and source**		**Brain defects**	**References**
Heterozygous LRRK2 (R1441G) transgenic mice + LPS	SNPC, MGCs^P/M++^ PBMCs/B cells^P/M++^	IFNγ	SNPC^P++^ Striatum^P+^ Sera^P+^	Neuron death	(120)
Heterozygous LRRK2 (R1441G) transgenic mice LRRK2^+/+^ + LPS	SNPC, MGCs^P/M++^ PBMCs/B cells^P/M++^	TNFα	MGCs^P/M(ND)^ SNPC^P/M(ND)^ Striatum^P+^ Sera^P++^	Neuron death Increased cognitive impairment	(91, 95, 120–122)
Heterozygous LRRK2 (R1441G) transgenic mice + LPS	SNPC, MGCs^P/M++^ PBMCs/B cells^P/M++^	IL-1α	SNPC^P/M+^ Striatum^P++^ Sera^P−^	Neuron death	(120)
Heterozygous LRRK2 (R1441G) transgenic mice LRRK2^+/+^ + LPS	SNPC, MGCs^P/M++^ PBMCs/B cells^P/M++^	IL-1β	MGCs^P/M(ND)^ Sera^P+^	Neuron death Increased cognitive impairment	(91, 95, 120–122)
Heterozygous LRRK2 (R1441G) transgenic mice + LPSLRRK2^+/+^ + LPS	SNPC, MGCs^P/M++^ PBMCs/B cells^P/M++^	IL-6	MGCs^P/M++^ SNPC^P++^ Striatum^P++^ Sera^P+^	Neuron death Increased cognitive impairment	(91, 95, 120–123)
Heterozygous LRRK2 (R1441G) transgenic mice	MGCs^P/M++^	IL-8	MGCs^P/M++^	Increased cognitive impairment	(1)
Heterozygous LRRK2 (R1441G) transgenic mice + LPS	SNPC, MGCs^P/M++^ PBMCs/B cells^P/M++^	IL-10	SNPC^P+^ Striatum^P++^ Sera^P++^	Neuron death	(120)
Heterozygous LRRK2 (R1441G) transgenic mice		IL-12	MGCs^P/M++^	Increased cognitive impairment	(91, 122)
Heterozygous LRRK2 (R1441G) transgenic mice	SNPC, MGCs^P/M++^ PBMCs/B cells^P/M++^	CCL2/MCP1	SNPC^P++^ Striatum^P++^	Neuron death	(120)
Heterozygous LRRK2 (R1441G) transgenic mice	SNPC, MGCs^P/M++^ PBMCs/B cells^P/M++^	CCL3/MIP1α	Striatum^P++^ S^P++^	Neuron death	(91, 120)
Heterozygous LRRK2 (R1441G) transgenic mice	MGCs^P++^	CCL4/MIP1β	MGCs^P/M++^	Neuron death	(91)
Heterozygous LRRK2 (R1441G) transgenic mice + LPS	SNPC, MGCs^P/M++^ PBMCs/B cells^P/M++^	CCL5/RANTES	Sera^P++^	Neuron death	(120)
Heterozygous LRRK2 (R1441G) transgenic mice	SNPC, MGCs^P/M++^ PBMCs/B cells^P/M++^	CXCL1/KC	SNPC^P+^ Striatum^P+^ Sera^P++^ MGCs^P/M++^	Neuron death	(91, 120)
Heterozygous LRRK2 (R1441G) transgenic mice	SNPC, MGCs^P/M++^ PBMCs/B cells^P/M++^	CXCL10	SNPC^P++^ Striatum^P+^ Sera^P−^	Neuron death	(120)
Heterozygous LRRK2 (R1441G) transgenic mice + LPS	SNPC, MGCs^P/M++^	GCSF	Sera^P++^	Neuron death	(120)
Heterozygous LRRK2 (R1441G) transgenic mice + LPS	SNPC, MGCs^P/M++^ PBMCs/B cells^P/M++^	MCSF	Sera^P++^	Neuron death	(120)

*LRRK2, leucine-rich-repeat kinase 2; IFN, interferon; TNF, tumor necrosis factor; TGF, transforming growth factor; IL, interleukin; α, alpha; β, beta; γ, gamma; CCL, chemokine C-C motif ligand; CXCL, chemokine C-X-C motif ligand; MCSF, macrophage colony-stimulating factor; GCSF, granulocyte colony-stimulating factor; SNPC, substantia nigra pars compacta; PBMCs, peripheral mononuclear cells; MGCs, microglial cells; M, mRNA expression; P, protein expression; ++, higher increases; +, moderate increases; –, decreased level; **∞**, no change; ND, no data*.

**Table 2B:** Cytokines and their source in the LRRK2-associated human PD.

**Human PD**	**LRRK2 kinase level and its source**	**Pro-inflammatory cytokines, chemokines, and their source**		**Brain defects**	**References**
LRKK2 G2019S mutation carriers	LRRK2^M++^	IFNγ	PBMCs^P−^	Increased cognitive impairment	(7)
LRKK2 G2019S mutation carriers	MO^P+^ T cells^P++^	TNFα	T cells^P+^ Sera^P++^	Neuron death Increased cognitive impairment	(91, 95, 121, 122, 124)
LRKK2 G2019S mutation carriers	MO^P+^ T cells^P++^	IL-1β	MO^P+^ Sera^P++^	Neuron death Increased cognitive impairment	(91, 95, 121, 122, 124)
LRKK2 G2019S mutation carriers	MO^P+^ T cells^P++^	IL-2	MO^P+^ T cells^±^	Neuron death	(124)
LRKK2 G2019S mutation carriers	MO^P+^ T cells^P++^	IL-4	MO^P+^ PBMCs^P++^	Neuron death	(120, 124)
LRKK2 G2019S mutation carriers	MO^P+^ T cells^P++^	IL-6	MO^P++^ PBMCs^P++^ Sera^P+^ CSF^P+^	Neuron death Increased cognitive impairment	(4, 120, 124)
LRKK2 G2019S mutation carriers	LRRK2^M++^	IL-8	Sera^P++^ CSF^P++^	Increased cognitive impairment	(122)
LRKK2 G2019S mutation carriers	LRRK2^M++^	IL-10	PBMCs^P++^ Sera^P++^	Increased cognitive impairment	(4)
LRKK2 G2019S mutation carriers	LRRK2^M++^ MO^P+^ T cells^P++^	IL-12	MO^P++^ T cells^P++^ Sera^P++^	Increased cognitive impairment	(91, 122, 124)
LRKK2 G2019S mutation carriers	MO^P+^ T cells^P++^	IL-13	MO^P++^ T cells^ND^	Increased cognitive impairment	(124)
LRKK2 G2019S mutation carrier	LRRK2^P++^	GCSF	Sera^P++^	Increased cognitive impairment	(4)
LRKK2 G2019S mutation carriers	LRRK2^P++^	PDGF	Sera^P++^	Increased cognitive impairment	(4)
LRKK2 G2019S mutation carriers	LRRK2^P++^	VEGF	CSF^P++^	Increased cognitive impairment	(91)

*LRRK2, leucine-rich-repeat kinase 2; IFN, interferon; TNF, tumor necrosis factor; TGF, transforming growth factor; IL, interleukin; α, alpha; β, beta; γ, gamma; CCL, chemokine C-C motif ligand; CXCL, chemokine C-X-C motif ligand; GCSF, granulocyte colony-stimulating factor; VEGF, vascular endothelial growth factor; PDGF, platelet-derived growth factor; LRRK2, leucine-rich-repeat kinase 2; PBMCs, peripheral mononuclear cells; MO, monocytes; CSF, cerebrospinal fluid; M, mRNA expression; P, protein expression; ++, higher increases; +, moderate increases; –, decreased level; **∞**, no change; ND, no data*.

## *GBA1* Gene Defects and Pro-inflammatory Immune Mediators in PD

The *GBA1* gene encodes the lysosomal enzyme, acid β-glucosidase (glucocerebrosidase, GCase). This later enzyme cleaves the β-D-glucosidic bond from the glycosphingolipid substrates (glucosylceramide; GC), yielding β-D-glucose and ceramide, and its deacylated product, glucosylsphingosine (GS), resulting in the formation of β-D-glucose and sphingosine (125, 126). The three types of Gaucher disease (GD), i.e., types 1, 2, and 3, have been characterized by recessive mutations in the *GBA1* gene. Pathogenic mutations in *GBA1* and the resultant GCase deficiency cause excess tissue accumulation of GC and chronic tissue inflammation in type 1 GD (59, 125, 127–133). We have identified immune complexes of GC-specific immunoglobulin G (IgG) antibodies in experimental and clinical Gaucher disease, which induce massive generation of complement C5a (C5a) and the activation of C5a receptor (e.g., C5aR1). Such C5a–C5aR1 activation is what tips the balance between GC formation and its degradation through the control of an enzyme termed as glucosylceramide synthase (GCS) that produces the GC and fuels inflammation in visceral tissues (e.g., blood, bone marrow, lung, liver, spleen, and lymph node) in type 1 experimental and clinical GD (131).

Excess brain accumulation of GC has been linked to the formation of abnormal species of α-syn, microglial cell activation, generation of pro-inflammatory cytokines (e.g., TNFα, IL-1β, and IL-6), and the loss of neurons in patients with GD types 2 and 3 (134–139). Heterozygous mutations in the *GBA1* gene are implicated in dementia with LBs (DLB) in idiopathic PD (140, 141). Similarly, the heterozygous *GBA1* mutations have emerged as the major genetic risk for developing PD (133, 138, 142–159).

Brains of the GBA1 mouse model of PD have shown partial GCase deficiency and its impact on increased production of TNFα, IL-1β, TGFβ1, CCL2, CCL3, CCL5, VCAM-1, ICAM-1, and MCSF as well as their link to the neuronal cell death (Table 3A). Plasma, sera, CSF, and blood-derived MOs of PD patients with *GBA* mutations have shown partial GCase deficiency and its impact on the higher production of pro-inflammatory cytokines (e.g., IFNγ, TNFα, IL-1β, IL-2, IL-4, IL-6, IL-8, IL-13, CCL2, CCL3, CCL18, and SF), midbrain damage, and cognitive defects (Table 3B). These studies suggest that GBA defects and the resultant GCase deficiency cause excess tissue storage of glycosphingolipids and/or the formation of abnormal species of α-syn. These abnormal proteins and/or lipids trigger residential and infiltrated immune cell (e.g., MOs and MGCs) activation and massive brain generation of pro-inflammatory cytokines and chemokines (Tables 3A,B), which are all critical for the development of brain inflammation and neurodegeneration in GBA-associated PD (Figure 1B).

**Table 3A:** Cytokines and their source in the mouse model of GBA1 PD.

**PD mouse model**	**GCase level and its source**	**Pro-inflammatory cytokines, chemokines, and their source**		**Brain defects**	**References**
Neuronopathic GBA^+/−^ GBA Het knock-in GBA^+/L444P^	Brain: >25% GCase VMB ^(P−)^	TNFα	Gray matter^P++^ Gray matter^M++^	BBB permeabilization, neuronal death	(135, 160)
Neuronopathic GBA^+/−^ GBA Het knock-in GBA^+/L444P^	Brain: >25% GCase VMB ^(P−)^	IL-1β	Gray matter^M++^	Neuronal cell death, BBB permeabilization	(160)
Neuronopathic GBA^+/−^ GBA Het knock-in GBA^+/L444P^	Brain: >25% GCase VMB ^(P−)^	TGFβ1	Gray matter^M++^	BBB permeabilization, neuronal death	(160, 161)
Neuronopathic GBA^+/−^ GBA Het knock-in GBA^+/L444P^	Brain: >25% GCase VMB ^(P−)^	CCL2/MCP1	Gray matter^M++^	BBB permeabilization, neuronal death	(135, 160)
Neuronopathic GBA^+/−^ GBA Het knock-in GBA^+/L444P^	Brain: >25% GCase VMB ^(P−)^	CCL3/MIP1α	Gray matter^M++^	BBB permeabilization, neuronal death	(135, 160)]
Neuronopathic GBA^+/−^ GBA Het knock-in GBA^+/L444P^	Brain: >25% GCase VMB ^(P−)^	CCL5/RANTES	Gray matter^M++^	BBB permeabilization, neuronal death	(135, 160)
NeuronopathicGBA^+/−^ GBA Het knock-in GBA^+/L444P^	Brain: >25% GCase VMB ^(P−)^	VCAM-1	ECs^M++^	BBB permeabilization, neuronal death	(135, 160)]
Neuronopathic GBA^+/−^ GBA Het knock-in GBA^+/L444P^	Brain: >25% GCase VMB ^(P−)^	ICAM-1	ECs^M++^	BBB permeabilization, neuronal death	(135, 160)
Neuronopathic GBA^+/−^ GBA Het knock-in GBA^+/L444P^	Brain: >25% GCase VMB ^(P−)^	MSCF	Gray matter^M++^	BBB permeabilization, neuronal death	(135, 160)

*GBA, acid β-glucosidase; GCase, glucocerebrosidase; GlcCer, glucosylceramide; IFN, interferon; TNF, tumor necrosis factor; TGF, transforming growth factor; IL, interleukin; α, alpha; β, beta; γ, gamma; CCL, chemokine C-C motif ligand; CXCL, chemokine C-X-C motif ligand; RANTES, regulated upon activation normal T-cell expressed and presumably secreted; MCSF, macrophage colony-stimulating factor; GCSF, granulocyte colony-stimulating factor; ICAM-1, intercellular adhesion molecule-1; VCAM-1, vascular cell adhesion molecule-1; MCP-1, monocyte chemoattractant protein-1; MIP1, macrophage inflammatory proteins; PARC, pulmonary and activation-regulated chemokine; SCF, stem cell factor; VMB, ventral midbrain; ECs, endothelial cells; M, mRNA expression; P, protein expression; ++, higher increases; +, moderate increases; –, decreased level; **∞**, no change; ND, no data*.

**Table 3B:** Cytokines and their source in the GBA-associated human PD.

**Human PD**	**GCase level and its source**	**Pro-inflammatory cytokines, chemokines, and their source**		**Brain defects**	**References**
GBA-linked PD	Plasma^GCase(−)^ MOs^GCase(−)^	IFNγ	Plasma^P++^	BBB leakage in the striatum and midbrain	(154, 162, 163)
	Plasma, CSF, nigrostriatal DA regions^GCase(−)^ MOs^GCase(−)^	TNFα	Plasma^P++^	BBB leakage in the striatum and midbrain	(154, 162, 163)
GBA-linked PD	Plasma, CSF, nigrostriatal DA regions MOs^GCase(−)^	IL-1β	Plasma^P++^	BBB leakage in the striatum and midbrain	(154, 162, 163)
GBA-linked PD	Plasma, CSF, nigrostriatal DA regions^GCase(−)^ MOs^GCase(−)^	IL-2	Plasma^P++^	BBB leakage in the striatum and midbrain	(154, 162, 163)
GBA-linked PD	CSF, nigrostriatal DA regions^GCase(ND)^ MOs^GCase(−)^	IL-4	Plasma ^P−^	BBB leakage in the striatum and midbrain	(154, 164)
GBA-linked PD	CSF, nigrostriatal DA regions^GCase(ND)^ MOs^GCase(−)^	IL-6	Plasma ^P++^	BBB leakage in the striatum and midbrain	(154, 165)
GBA-linked PD	Plasma >25% GCase MOs^GCase(−)^	IL-8	Plasma^P++^	Cognitive dysfunction	(154, 161)
GBA-linked PD	Plasma >25% GCase MOs^GCase(−)^	IL-13	Plasma^P++^	BBB leakage in the striatum and midbrain	(154, 162)
GBA-linked PD	Plasma: >25% GCase MOs^GCase(−)^	CCL2/MCP-1	Plasma^P+^	Cognitive dysfunction	(154, 161)
GBA-linked PD	Plasma: >25% GCase MOs^GCase(−)^	CCL3/MIP1α	Plasma^P++^	Cognitive dysfunction	(154, 161)
GBA-linked PD	Plasma: >25% GCase MOs^GCase(−)^	CCL18/PARC	Plasma^P++^	Cognitive dysfunction	(154, 161)
GBA-linked PD	Plasma: >25% GCase MOs^GCase(−)^	SCF	Plasma^P−^	Cognitive dysfunction	(154, 161)

*GBA, acid β-glucosidase; GCase, glucocerebroside; IFN, interferon; TNF, tumor necrosis factor; TGF, transforming growth factor; IL, interleukin; α, alpha; β, beta; γ, gamma; CCL, chemokine C-C motif ligand; CXCL, chemokine C-X-C motif ligand; VCAM-1, vascular cell adhesion molecule 1; MCP-1, monocyte chemoattractant protein-1; MIP1, macrophage inflammatory protein; PARC, pulmonary and activation-regulated chemokine; SCF, stem cell factor; CSF, cerebrospinal fluid; DA, dopaminergic; PBMCs, peripheral mononuclear cells; BMVECs, brain microvessel endothelial cells; BBB, blood–brain barrier; Mon, monocytes; M, mRNA expression; P, protein expression; ++, higher increases; +, moderate increases; –, decreased level; **∞**, no change; ND, no data*.

## *SNCA* gENE Defects and Pro-inflammatory Immune Mediators in PD

*SNCA* encodes the α-syn, which is an 18-kDa protein composed of 140 amino acids and expressed in presynaptic terminals of the neocortex, hippocampus, substantia nigra (SN), NCs, ACs, and oligodendrocytes as well as CSF, serum, plasma, and hematopoietic cells (166–173). The brain α-syn interacts with proteins and lipids and controls the synaptic vesicle recycling and neurotransmitter release (174–177). However, the SNCA defect and the resultant excess generation and/or formation of normal endogenous or aggregated Agg α-syn in cytoplasmic inclusions of NCs termed as LBs and Lewy neurites (LNs) lead to neuronal toxicity and neurodegeneration in early- and late-onset PD (166, 178–185). Strikingly, LBs and LNs of the idiopathic forms of PD have also shown excess of α-syn and the Agg α-syn without any *SNCA* mutation (183, 186–188). In contrast, overexpression of wild-type SNCA and the resultant higher production of WT α-syn show their link to neurotoxicity in *Drosophila melanogaster* (189) and rodent models (190). Normal and Agg α-syn have shown TLR2- or TLR4-mediated MGC activation and neuronal loss in PD and mouse models (70, 191–198). PD genome-wide association studies (GWAS) identified the risk variants in certain loci associated to disease risk such as HLA-DR locus, which encodes for the major histocompatibility complex I (MHC class II) known for triggering the antigen presentation to CD4^+^ T cells (199–202). Two classical pathways of antigen presentation have been described for the presentation of endogenous antigens on MHC I molecules and the presentation of exogenous antigens, such as intracellular pathogens, on MHC class II molecules [reviewed by Blum et al. (203)]. The MHCII pathway is performed by specialized antigen-presenting cells, i.e., Mϕs, DCs, and DA neurons, which present peptides on MHCII molecules, ensuring its efficient recognition by CD4^+^ T cells (204). In addition to the increased brain infiltration of effector T-cell subsets in PD patients (42, 43), MHCII-mediated presentation of α-syn to CD4^+^ T cells has been linked to neuroinflammation in a mouse model and human PD (205–207). α-Syn peptide-stimulated T cells have shown development of activated subsets of helper and cytotoxic T cells and increased production of IFNγ, IL-2, and IL-5 (205). In addition, one of the peptide regions strongly binds to MHC encoded by HLA (DRB1^*^15:01, DRB5^*^01:01) linked to PD by GWAS (201, 208–210).

The sera, MGCs, and brain regions of the SNCA mouse model of PD have shown overexpression of different species of α-syn and pro-inflammatory cytokines (e.g., IFNγ, TNFα, IL-1α, IL-1β, IL-6, IL-10, TGFβ, CCL2, CCL3, CCL5, CXCL10, and ICAM-1) as well as their link to neuronal cell death and cognitive defects (Table 4A). The blood-derived immune cells, sera, and brain regions of PD patients with SNCA defect have also shown overexpression of α-syn and their association with cellular activation and increased generation of pro-inflammatory mediators (e.g., IFNγ, TNFα, IL-1β, IL-4, IL-5, IL-6, IL-18, and CCL2) as well as their link to neuronal cell damage (Table 4B). Hence, SNCA defects and the resultant increased making of normal and/or Agg α-syn promote the activation of peripheral immune cells and the brain MGCs. Such cells cause massive generation of NO, ROS, and pro-inflammatory cytokines and chemokines (Tables 4A,B), which are all critical for promoting brain inflammation and neurodegeneration in SNCA-associated PD (Figure 1C).

**Table 4A:** Cytokines and their source in the mouse model of SNCA PD.

**PD mouse model**	**α-Syn and its source**	**Pro-inflammatory cytokines, chemokines, and their source**		**Brain defects**	**References**
Local rAAV-A53T-α-syn injection in WT mice SN Aggregated α-syn-stimulated WT microglial cells MHCII/HLA-mediated activation of DC and CD4^+^ T cells of D409/D409 murine model	^P/M++^	IFNγ	Striatum^P+^ SNPC^M−^	Neuron death	(205–207, 211–213)
Thy-1 α-syn overexpression murine model of PD A53T α-syn over-expressing SHSY5Y cells Astrocytoma cell line U373 engineered to express C-terminally truncated α-syn Local rAAV-A53T-α-syn injection in WT mice SN α-Syn-stimulated DM-A30P–A53T microglial cells Aggregated α-syn-stimulated WT microglial cells rα-syn-stimulated WT; A53T; A30P; E46K macrophages Monomeric α-syn-stimulated WT; A53T rat primary microglial cells WT, A53T α-syn overexpressing SHSY5Y microglial cell lines N-α-syn-stimulated WT microglial cells Syn-stimulated microglial cell lines (BV2) MHCII-mediated activation of DC and CD4^+^ T cells of D409/D409 murine model α-Syn-injected (intra-SN) ABH Biozzi mice	SNPC^P++^ Striatum^P++^ Cortex^P++^ SNCA^P/M++^ ND ^P++^ ^P++^ ND ND ND ND ND ND ND	TNFα	Striatum^P++^ SNPC^P++^ Cortex^P++^ Serum^P++^ MGCs^M/P++^ Striatum^P+^ CC^P+^ Striatum^M+^ SNPC^M+^ CC^M/P+^ SNPC^M+^ MGCs^P++^ SNPC^M++^	Neuron death Increased cognitive impairment	(43, 191, 192, 197, 198, 206, 207, 211–222)
Thy-1 α-syn overexpression murine model of PD Local rAAV-A53T-α-syn injection in WT mice SN WT, A53T α-syn overexpressing SHSY5Y microglial cell lines	^P++^ ^P/M++^ ND	IL-1α	MGCs^M++^ SNPC^M++^ SNPC^M/P++^ MGCs^M/P++^		(192, 213, 215)
Thy-1 α-syn overexpression murine model of PD A53T α-syn overexpressing SHSY5Y cells α-Syn-injected (intra-SN) ABH Biozzi mice Local rAAV-A53T-α-syn injection in WT mice SN α-syn-stimulated DM-A30P–A53T microglial cells α-Syn-stimulated WT microglial cells Syn-stimulated microglial cell lines (BV2) N-α-syn-stimulated WT microglial cells Monomeric α-syn-stimulated WT; A53T; A30P; E46K microglial cells Monomeric α-syn-stimulated WT; A53T rat microglial cells WT, A53T α-syn overexpressing SHSY5Y	^P/M++^ Striatum^P++^ Cortex^P++^ ^P/M++^ ^P/M++^	IL-1β	Striatum^P−^ SNPC^P−^ Cortex^P−^ Serum^P−^ MGCs^M++^ SNPC^M/P++^ MGCs^M/P++^ Striatum^P+^ SNPC^P++^ MGCs^M++^ Striatum^M+^ SNPC^M++^ CC^M/P++^ MGCs^P+^ MGCs^P+^	Neuron death Increased cognitive impairment	(191, 192, 197, 211, 216, 218–220, 222, 223)
Thy-1 α-syn overexpression murine model of PD A53T alpha-synuclein overexpressing SHSY5Y cells Local rAAV-A53T-α-syn injection in WT mice SN α-syn I -stimulated DM-A30P–A53T microglial cells N-α-syn-stimulated WT microglial cells Aggregated α-syn-stimulated WT microglial cells Monomeric α-syn-stimulated WT; A53T; A30P; E46K microglial cells a-Syn-stimulated microglial cell lines (BV2) Transient transfection in microglial cell lines MHCII-mediated activation of DC and CD4^+^ T cells of D409/D409 murine model Monomeric α-syn-stimulated WT; A53T; A30P	^P++^ ^P/M++^ ND ND ND ND ND ND ND ND	IL-6	SNPC^M++^ SNPC^P/M++^ Striatum^M+^ SNPC^M+^ MGCs^P++^ MGCs^P++^ MGCs^P++^	Neuron death	(191, 192, 206, 207, 212, 215, 216, 218, 220, 221)
Monomeric α-syn stimulated WT; A53T; A30P; E46K microglial cells	^P/M++^	IL-10	Striatum^M+^ SNPC^M+^ MGCs^P++^	Neuron death	(38, 216, 222)
Monomeric α-syn-treated mice	^P++^	TGFβ	SNPC^M++^	Neuron death	(197, 213)
Aggregated α-syn-stimulated WT microglial cells Monomeric α-syn-stimulated WT; A53T; A30P; E46K microglial cells A53T alpha-synuclein overexpressing SHSY5Y cells	^P++^	CCL2/MCP1	MGC^P++^	Neuron death	(212, 218, 220)
Monomeric α-syn-stimulated WT; A53T; A30P; E46K microglial cells	MGCs^P++^	CCL3/MIP1α	MGCs^P++^	Neuron death	(38)
Monomeric α-syn-stimulated WT; A53T; A30P; E46K microglial cells	MGCs^P++^	CCL5/RANTES	MGCs^P++^	Neuron death	(220)
Mutant α-syn overexpression murine model of PD	^P/M++^	CX3CR1	Striatum^M+^ SNPC^M+^	Neuron death	(216)
A53T; A30P; E46K microglial cells	^P/M++^	CXCL10	MGCs^M/P++^	Neuron death	(222)
Local AAV α-syn overexpression murine model of PD	^P++^	ICAM-1	SNPC^M+^	Neuron death	(215)

*SNCA, synuclein alpha; IFN, interferon; TNF, tumor necrosis factor; TGF, transforming growth factor; IL, interleukin; α, alpha; β, beta; γ, gamma; CCL, chemokine C-C motif ligand; CXCL, chemokine C-X-C motif ligand; CX3CR1, chemokine C-X-R receptor; RANTES, regulated upon activation, normal T-cell expressed and presumably secreted; ICAM-1, intercellular adhesion molecule-1; MCP-1, monocyte chemoattractant protein-1; MIP1α, macrophage inflammatory proteins; SNPC, substantia nigra pars compacta; TC, T cells; MGCs, microglial cells; BG, basal ganglia; M, mRNA expression; P, protein expression; ++, higher increases; +, moderate increases; –, decreased level; **∞**, no change; ND, no data*.

**Table 4B:** Cytokines and their source in the SNCA-associated human PD.

**Human PD**	**α-Syn and its source**	**Pro-inflammatory cytokines, chemokines, and their source**		**Brain defects**	**References**
PD patients' T cells	^P++^	IFNγ	TC^P++^ SNPC^P+^ BG^P+^	Damaging of dopaminergic neurons	(205–207, 211, 212)
U373 cells overexpressing truncated α-synuclein: PD patients' brain	^P++^	TNFα	SNPC^P+^ MGCs^M+^	Damaging of dopaminergic neurons	(122, 192, 197, 198, 206, 207, 211, 212, 214–219)
PD patients' brain Sera	Brain^P++^ Sera^P++^	IL-1β	Sera^P+^ MGCs^M+^ PBMCs^P++^	Damaging of dopaminergic neurons	(61, 122, 191, 192, 197, 211, 216, 218–220, 223)
PD patients' T cells	^P++^	IL-4	TC^P++^	Damaging of dopaminergic neurons	(205)
PD patients' T cells	^P++^	IL-5	TC^P++^	Damaging of dopaminergic neurons	(205)
PD patients' brain Sera	Brain^P++^ Sera^P++^	IL-6	SNPC^P+^ MGCs^M+^ BG^P+^ PBMCs^P++^	Damaging of dopaminergic neurons	(61, 122, 191, 192, 206, 207, 212, 215, 216, 218, 220, 221)
Patient sera	Sera^P++^	IL-18	PBMCs^P++^	Damaging of dopaminergic neurons	(61)
PD patients' brain	Brain^P++^	CCL2/MCP1	SNPC^P+^ MGCs^M+^ BG^P+^	Damaging of dopaminergic neurons	(212, 218, 220)

*SNCA, synuclein alpha; IFN, interferon; TNF, tumor necrosis factor; TGF, transforming growth factor; IL, interleukin; α, alpha; β, beta; γ, gamma; MCP-1, monocyte chemoattractant protein-1; SNPC, substantia nigra pars compacta; TC, T cells; MGCs, microglial cells; BG, basal ganglia; ECs, endothelial cells; M, mRNA expression; P, protein expression; ++, higher increases; +, moderate increases; –, decreased level; **∞**, no change; ND, no data*.

## *PARK2* Gene Defects and Pro-inflammatory Immune Mediators in PD

The *PARK2* gene encodes cytosolic ubiquitin E3 ligase termed as parkin protein, which is critical for the targeting, breakdown, and recycling of damaged proteins as well as the regulation of mitophagy and survival of DA neurons (224). *PARK2* mutations cause a loss of parkin function that leads to the excess accumulation of dysfunctional mitochondria and the resultant massive generation of oxidative stress and death of DA neurons in autosomal recessive and idiopathic PD (225–235). CD4^+^ and CD8^+^ cell infiltration, MGC activation, increased generation of pro-inflammatory cytokines, and the loss of DA neurons have been observed in mouse model and human PD (43, 236).

Parkin plays a protective role during bacterial and viral infection and chemically induced oxidative and ER stress by altering the mitochondrial ROS and pro-inflammatory cytokine-mediated downstream signaling cascades (237–247). Biochemical and genetic studies reveal that parkin also acts in tandem with phosphatase and tensin homolog (PTEN)-induced putative kinase 1 (PINK1), which is accountable for controlling the mitochondrial quality (248). Indeed, mutations in the genes that encode PINK1 and Parkin showed massive mitochondrial damage and the development of familial PD (229). It has been shown that autophagy, the recycling of self-components through lysosomal degradation, is involved in the presentation of endogenous antigens on both MHC class I and class II molecules (249, 250), highlighting that vacuolar content can also be presented on MHC class I/II molecules. The mitochondrial MHCI-mediated antigen processing and presentation to CD8^+^ T cells have been valued for induction of neuroinflammation in mouse models and human PD (42, 43, 205, 251, 252). To understand the exact role of parkin and PINK1 in the development of brain inflammation in PD, Matheoud et al. (252) have discovered a pathway for mitochondrial antigen presentation, in which mitochondria-derived vesicles targeted endolysosomes for processing and presentation by MHC class I molecules. Using both *in vitro* and *in vivo* experiments, this study has demonstrated that parkin and PINK1 inhibit mitochondria-derived vesicle formation and mitochondrial antigen presentation, and therefore, in the absence of PINK1 or parkin, mitochondrial antigen presentation triggers DC and CD8^+^ T-cell activation and increased generation of pro-inflammatory cytokines. These data suggest that PINK1 and/or parkin has a key role in the activation of innate and adaptive immune cells by repressing the presentation of mitochondrial antigens, which suggests the involvement of autoimmune reactions in PD (252). *PARK2* mutations and their link to α-syn inclusions and LB formation have also been observed in exceptional cases of PARK2-associated PD (253–255). The exact mechanism by which PARK2 defects propagate brain inflammation and neurodegeneration in PD is poorly defined.

The MGCs, Mϕs, and sera of the PARK2 mouse model displayed decreased expression of parkin and its link to the increased generation of pro-inflammatory cytokines and chemokines (e.g., IFNβ1, TNFα, IL-1β, IL-12, IL-13, IL-17, CCL2, and CXCL1), loss of DA neurons, and cognitive defects in PD (Table 5A). The sera, MGCs, Mϕs, and midbrain regions of PARK2-associated human PD also displayed decreased expression of parkin and its link to increased generation of pro-inflammatory cytokines (e.g., IFNβ1, TNFα, IL-1β, IL-6, IL-12, IL-13, CCL2, CCL4, and CXCL1), loss of DA neurons, and cognitive defects in PD (Table 5B). These findings suggest that PARK2 and the resultant deficiency of parkin are associated with mitochondrial damage and/or the formation of Agg α-syn. These defects cause cellular activation and massive generation of pro-inflammatory cytokines and chemokines (Tables 5A,B), which lead to the loss of DA neurons in PARK2-associated PD (Figure 1D).

**Table 5A:** Cytokines and their source in the mouse model of PARK2 PD.

**PD mouse model**	**Parkin level and its source**	**Pro-inflammatory cytokines, chemokines, and their source**		**Brain defects**	**References**
SED Parkin^−/−^, Parkin^+/−^	Sera^M−*andP*−^ Sera^P−^	IFNβ1	Sera^P+^ Sera^P+^	Death of DA neurons in SNPC and motor defects	(79)
Parkin^−/−^ and WT mice	Midbrain^M−*andP*−^ Cortex^M−*andP*−^ Mφs^M−^ MGCs^M−^	TNFα	Midbrain^M+^ Cortex^ND^ Mφs^M+^ MGCs^M+^	Nigral cell degeneration and DA loss in SNPC	(256, 257)
Parkin^−/−^ and WT mice	Mφs^M−^ MGCs^M−^	IL-1β	Mφs^M+^ MiGCs^M+^	Nigral cell degeneration and DA loss in SNPC Loss of fine motor skills	(257, 258)
SED Parkin^−/−^, Parkin^+/−^	Sera^M−*andP*−^ Sera^P−^	IL-12	Sera^P+^ Sera^P+^	Death of DA neurons in SNPC and motor defects	(79)
SED Parkin^−/−^, Parkin^+/−^	Sera^M−*andP*−^ Sera^P−^	IL-13	Sera^P+^ Sera^P+^	Death of DA neurons in SNPC and motor defects	(79)
SED Parkin^−/−^, Parkin^+/−^	Sera^M−*andP*−^ Sera^P−^	IL-17	Sera^P+^ Sera^P+^	Death of DA neurons in SNPC and motor defects	(79)
SED Parkin^−/−^, Parkin^+/−^	Sera^M−*andP*−^ Sera^P−^	CCL2/MCP1	Sera^P+^ Sera^P+^	Death of DA neurons in SNPC and motor defects	(79)
SED Parkin^−/−^, Parkin^+/−^	Sera^M−*andP*−^ Sera^P−^	CXCL1/KC	Sera^P+^ Sera^P+^	Death of DA neurons in SNPC and motor defects	(79)

*IFN, interferon; TNF, tumor necrosis factor; TGF, transforming growth factor; IL, interleukin; α, alpha; β, beta; CXCL, chemokine C-X-C motif ligand; CCL, chemokine C-C motif ligand; MGCs, microglial cells; Mφs, macrophages; M, mRNA expression; P, protein expression; ++, higher increases; +, moderate increases; –, decreased level; **∞**, no change; ND, no data*.

**Table 5B:** Cytokines and their source in the PARK2-associated human PD.

**Human PD**	**Parkin level and its source**	**Pro-inflammatory cytokines, chemokines, and their source**		**Brain defects**	**References**
Parkin^+/−^ unaffected PD patients	Sera^P−^	IFNβ1	Sera^P+^	Inflammation, motor defects, and loss of DA neurons in SNPC	(79)
PARK2/Parkin-associated PD	Mφs^M−^ MGCs ^M−^	TNFα	Mφs^M+^ MGCs^M+^	Inflammation, motor defects, and loss of DA neurons in SNPC	(79)
PARK2/Parkin-associated PD	Mφs^M−^ MGCs^M−^	IL-1β	Mφs^M+^ MGCs^M+^	Motor deficits, loss of DA neurons in SNPC, inflammation-related nigral degeneration	(256, 257)
PD patients with biallelic PRKN/PINK1 mutations		IL-6	Sera^P+^	Mitophagy dysfunction	(259)
Parkin^+/−^ unaffected PD patients	Sera^P−^	IL-12	Sera^P+^	Mitophagy dysfunction and neuroinflammation	(259)
Parkin^+/−^ unaffected PD patients	Sera^P−^	IL-13	Sera^P+^	Inflammation, motor defects, and loss of DA neurons in SNPC	(79)
Parkin^+/−^ unaffected PD patients	Sera^P−^	CCL2/MCP1	Sera^P+^	Inflammation, motor defects, and loss of DA neurons in SNPC	(79)
Parkin^+/−^ unaffected PD patients	Sera^P−^	CCL4/MIP1β	Sera^P+^	Inflammation, motor defects, and loss of DA neurons in SNPC	(79)
Parkin^+/−^ unaffected PD patients	Sera^P−^	CXCL1/KC	Sera^P+^	Inflammation, motor defects, and loss of DA neurons in SNPC	(79)

*IFN, interferon; TNF, tumor necrosis factor; TGF, transforming growth factor; IL, interleukin; α, alpha; β, beta; CXCL, chemokine C-X-C motif ligand; CCL, chemokine C-C motif ligand; MGCs, microglial cells; Mφs, macrophages; M, mRNA expression; P, protein expression; ++, higher increases; +, moderate increases; –, decreased level; **∞**, no change; ND, no data*.

## *PARK6* Gene Defects and Pro-inflammatory Immune Mediators in PD

The *PARK6* gene encodes PINK1, which is a universally expressed serine/threonine kinase with a mitochondrial targeting sequence that directs the import of PINK1 as well as the activation and recruitment of parkin into the mitochondria for clearance of damaged mitochondria (260–267). PINK1-deficient cells, including NCs, are more susceptible to various insults (268, 269). PINK1 and parkin control the degradation of dysfunctional mitochondria (270, 271). PARK6 defects and the resultant deficiency of PINK1 lead to mitochondrial dysfunctions and the development of autosomal recessive and early-onset PD (261, 272–274). *Pink1*-deficient *Drosophila* displayed mitochondrial damage associated with apoptotic muscle degeneration and DA neuron loss, whereas Parkin overexpression protected such PINK1-induced defects (248, 275, 276). Several studies have shown that PINK1, like parkin, modulates NF-κB activity and brain generation of pro-inflammatory cytokines (277). PINK1-deficient T cells have reduced protein kinase B (PKB or Akt) activity, which is critical for inducible regulatory T cells (iTreg) development (278). PINK1-deficient iTreg cells showed reduced capacity to suppress lymphocyte proliferation (278). Importantly, the autologous transfer of Treg cells to MPTP-treated mice attenuated MGC activation and provides neuroprotection (279).

Strikingly, Treg cells from PD patients also have impaired suppressor function (47). T-cell subset infiltration and their interaction with MGCs and DA neurons are critical for the development of neuroinflammation and neurodegeneration in MPTP-induced mouse model and human patients with PD (43, 47, 48, 280, 281). Gram-negative bacteria-induced intestinal infection in Pink1^−/−^ mice showed mitochondrial antigen presentation to CD8^+^ T cells in the periphery and in the brain and their link to loss of DA axonal varicosities in the striatum and the motor impairment. These data suggest the relevance of the gut–brain axis that could develop brain inflammation and neurodegeneration in PD (282, 283).

The blood, brain regions, and cells of the mouse model of PARK6-associated PD have shown PINK1 deficiency and its impact on increased blood or brain generation of pro-inflammatory cytokines and chemokines (e.g., IFNγ, IFNβ1, TNFα, IL-1β, IL-2, IL-6, IL-10, IL-12, IL-13, IL-17, TGFβ, CCL2, CCL4, and CXCL1), loss of neuronal cells, and the development of cognitive defects in PD (Table 6A). Additionally, PARK6-associated PD patients have also shown PINK1 deficiency and its impact on increased generation of pro-inflammatory cytokines and chemokines (e.g., IFNβ1, IL-6, IL-12, IL-13, CCL2, CCL4, and CXCL1), loss of NCs, and the development of cognitive defects (Table 6B). These findings suggest that PARK6 and the resultant PINK1 defects trigger residential and infiltrated immune cell activation and increased production of pro-inflammatory cytokines and chemokines (Tables 6A,B), which ultimately lead to the loss of DA neurons in PARK6-associated PD (Figure 1E).

**Table 6A:** Cytokines and their source in the mouse model of PARK6 PD.

**PD mouse model**	**PINK1 level and its source**	**Pro-inflammatory cytokines, chemokines, and their source**		**Brain defects**	**References**
PINK1^−/−^	Striatal varicosities^P−^	IFNγ	Cytotoxic T cells^P+^	Motor impairment and loss of DA neurons in striatum varicosities	(282)
SED PINK1^−/−^ and ^+/−^	Sera^P−^	IFNβ1	Sera^P+^	Inflammation, motor defects, and loss of DA neurons in SNPC	(79)
PINK1^−/−^	Striatum^M−*andP*−^ MGCs^M−^ Astrocytes^M−^ Cortex^M−*andP*−^	TNFα	Striatum^M+^ MGCs^M−^ ACs^M+^ Cortex^M+andP+^	Inflammation-induced DA death. Disruption of DA neuron dysfunction	(258, 284, 285)
PINK1^−/−^	Striatum^M−*andP*−^ MGCs^M−^ Astrocytes^M−^ Cortex^M−*andP*−^	IL-1β	Striatum^M+^ MGCs^M−^ ACs^M+^ Cortex^M+andP+^	Inflammation-induced DA neuronal death	(258, 284, 285)
PINK1^−/−^	Striatal varicosities^P−^	IL-2	Cytotoxic T cells^P+^	Motor impairment and loss of DA neurons in striatum varicosities	(282)
PINK1^−/−^	Striatum^M−*andP*−^ Cortex^M−*andP*−^	IL-6	Striatum^P+^ Cortex^M+andP+^	Inflammation-induced DA neuronal death. Disruption of DA neuron dysfunction	(258, 277, 284, 285)
PINK1^−/−^	Striatum^M−*andP*−^ MGCs^M−^ Cortex^M−*andP*−^	IL-10	Striatum^P+^ MGCs^M−^ Cortex^M+andP+^	Inflammation-induced DA neuronal death. Disruption of DA neuron dysfunction	(258, 277, 284, 285)
PINK1^−/−^ and ^+/−^	Striatum^M−*andP*−^ Sera^P−^	IL-12	Striatum^P+^ Sera^P+^	Inflammation-induced DA neuronal death. Disruption of DA neuron dysfunction	(79, 258, 277)
SED PINK1^−/−^ and ^+/−^	Sera^P−^	IL-13	Sera^P+^	Inflammation, motor defects, and loss of DA neurons in SNPC	(79)
SED PINK1^−/−^ and ^+/−^	Sera^P−^	IL-17	Sera^P+^	Inflammation, motor defects, and loss of DA neurons in SNPC	(79)
PINK1^−/−^	Microglia^M−^ Astrocytes^M−^	TGFβ	MGCs^M−^ ACs^M+^	Inflammation-induced DA death	(284)
SED PINK1^−/−^ and ^+/−^	Sera^P−^	CCL2/MCP1	Sera^P+^	Inflammation, motor defects, and loss of DA neurons in SNPC	(79)
SED PINK1^−/−^ and ^+/−^	Sera^P−^	CCL4/MIP1β	Sera^P+^	Inflammation, motor defects, and loss of DA neurons in SNPC	(79)
SED PINK1^−/−^ and ^+/−^	Sera^P−^	CXCL1/KC	Sera^P+^	Inflammation, motor defects, and loss of DA neurons in SNPC	(79)

*PINK1; PTEN-induced kinase 1; IFN, interferon; TNF, tumor necrosis factor; TGF, transforming growth factor; IL, interleukin; α, alpha; β, beta; γ, gamma; CXCL, chemokine C-X-C motif ligand; CCL, chemokine C-C motif ligand; MGCs, microglial cells; Mφs, macrophages; M, mRNA expression; P, protein expression; ++, higher increases; +, moderate increases; –, decreased level; **∞**, no change; ND, no data*.

**Table 6B:** Cytokines and their source in the PARK6-associated human PD.

**Human PD**	**PINK1 level and its source**	**Pro-inflammatory cytokines/chemokines**	**Cytokines, chemokines, and their source**	**Brain defects**	**References**
PARK6/PINK1-associated PD	Sera^P−^	IFNβ1	Sera^P+^	Loss of DA neurons and motor defects	(79)
PARK6/PINK1-associated PD	Sera^P−^	IL-6	Sera^P+^	Cortical injuries and neuronal death	(259)
PARK6/PINK1-associated PD	Sera^P−^	IL-12	Sera^P+^	Cortical injuries and neuronal death	(79)
PARK6/PINK1-associated PD	Sera^P−^	IL-13	Sera^P+^	Loss of DA neurons and motor defects	(79)
PARK6/PINK1-associated PD	Sera^P−^	CCL2/MCP1	Sera^P+^	Loss of DA neurons and motor defects	(79)
PARK6/PINK1-associated PD	Sera^P−^	CCL4/MIP1β	Sera^P+^	Loss of DA neurons and motor defects	(79)
PARK6/PINK1-associated PD	Sera^P−^	CXCL1/KC	Sera^P+^	Loss of DA neurons and motor defects	(79)

*PINK1, PTEN-induced kinase 1; IFN, interferon; IL, interleukin; β, beta; CXCL, chemokine C-X-C motif ligand; CCL, chemokine C-C motif ligand; DA, dopaminergic; M, mRNA expression; P, protein expression; ++, higher increases; +, moderate increases; –, decreased level; **∞**, no change; ND, no data*.

## ***PARK7*** gENE Defects and Pro-inflammatory Immune Mediators in PD

*PARK7* encodes a protein deglycase DJ-1, which belongs to the peptidase C56 family of proteins and ubiquitously expressed under physiological conditions (286). Like PINK1 and parkin, DJ-1 is required for controlling mitochondrial damage and production of oxidative stress (287–289). Several chemicals and physiological factors trigger the upregulation of DJ-1, which protects the oxidative and endoplasmic reticulum stress-induced damage of endothelial cells, Mϕs, fibroblast, NCs, and islet β cells (290–296), and therefore, DJ-1 deficiency has been associated with the development of several diseases (e.g., stroke, male infertility, cancers, diabetes, and neurodegenerative illnesses) (290, 297, 298). *Escherichia coli-* or *Pseudomonas aeruginosa*-mediated excess activation of MAPK signaling and the resultant induction of brain inflammation have been observed in DJ-1-deficient *Caenorhabditis elegans* (299). Mutations in PARK7 and the resultant deficiency or the oxidized form of DJ-1 protein cause autosomal recessive early-onset and idiopathic PD as reviewed in ref. (300).

Brain regions and their cells of the mouse model of PARK7-associated PD have shown DJ-1 deficiency and its effect on increased production of IFNγ, IL-1β, IL-1Ra, IL-6, IL-16, IL-17, CXCL11, and NGF as well as on the damage of ACs and DA neurons (Table 7A). Furthermore, abnormal cellular and brain region expression of DJ-1 has been associated with the formation of α-syn and Tau containing LBs, mitochondrial damage, increased production of ROS, and their link to the loss of NCs in PD patients with *PARK7* mutation (Table 7B). These data suggest that PARK7 and the resultant DJ-1 deficiency induced mitochondrial damage and/or the formation of Agg α-syn and Tau comprising LB. These abnormal proteins cause massive generation of pro-inflammatory cytokines and chemokines (Tables 7A,B), which ultimately lead to the death of DA neurons in PARK7-associated PD (Figure 1F).

**Table 7A:** Cytokines and their source in the mouse model of PARK7 PD.

**PD mouse model**	**DJ-1 level and its source**	**Pro-inflammatory cytokines, chemokines, and their source**		**Brain defects**	**References**
DJ-1–/–	SN^M− *and P*−^	IFNγ	SN^P+^	Loss of DA neurons in the nigrostriatal pathway and striatal dopamine	(301)
DJ-1–/–, DJ-1 knockdown (shRNA)	SN^M− *and P*−^ MGCs^M−^	IL-1β	SN^M− *and P*−^ MGCs^P+^	Inflammation induced DA neuronal death. Loss of DA neurons in the nigrostriatal pathway and striatal dopamine	(258, 301–303)
DJ-1–/–	SN^M− *and P*−^	IL-1Ra	SN^P+^	Loss of DA neurons in the nigrostriatal pathway and striatal dopamine	(301)
DJ-1–/–, DJ-1 knockdown (shRNA)	MGCs^M−^ ACs^M−*andP*−^	IL-6	MGCs^P+^ ACs^P+^	Increased DA neurotoxicity. Deregulation of astrocytic neuroinflammatory damage	(302–304)
DJ-1–/–	SN^M− *and P*−^	IL-16	SN^P+^	Loss of DA neurons in the nigrostriatal pathway and striatal dopamine	(301)
DJ-1–/–	SN^M− *and P*−^	IL-17	SN^P+^	Loss of DA neurons in the nigrostriatal pathway and striatal dopamine	(301)
DJ-1–/–	SN^M− *and P*−^	CXCL11	SN^P+^	Loss of DA neurons in the nigrostriatal pathway and striatal dopamine	(301)
DJ-1–/–	ACs^M− *and P*−^	NGF	ACs^P+^	Deregulation of astrocytic neuroinflammatory damage	(304)

*DJ-1, protein deglycase-1; IFN, interferon; IL, interleukin; ILRa, interleukin receptor antagonist; α, alpha; β, beta; γ, gamma; CXCL, chemokine C-X-C motif ligand; CCL, chemokine C-C motif ligand; NGF, nerve growth factor; SN, substantia nigra; MGCs, microglial cells; ACs, astrocytes; shRNA, short hairpin ribonucleic acid; DA, dopaminergic; M, mRNA expression; P, protein expression; ++, higher increases; +, moderate increases; –, decreased level; **∞**, no change; ND, no data*.

**Table 7B:** Cytokines and their source in the PARK7-associated human PD.

**Human PD**	**DJ-1 level and its source**	**Pro-inflammatory cytokines, chemokines, and their source**		**Brain defects**	**References**
PARK7/DJ-1-associated PD	Alpha synuclein in SNPC^P−^ DJ-1 in HEK293 cells^M−*andP*−^ DJ-1 in substantia nigra^P+^ Oxidized DJ-1 in Lewy bodies^P+^ Oxidized DJ-1 in astrocytes^P+^ DJ-1 and Tau protein in neurofibrillary tangles^P+^ Postmortem full brain^M−*andP*−^	ND	ND	Loss of DA neurons in SNPC, Lewy body formation, motor defects, muscle wasting NO-induced DA neuronal	(300, 305)

*Of special note, no definite or concrete data have been found about cytokine levels in PARK7-human associated PD*.

*DJ-1, protein deglycase-1; SNPC, substantia nigra pars compacta; HEK293, human embryonic kidney-293; NO, nitric oxide synthase; DA, dopaminergic; M, mRNA expression; P, protein expression; ++, higher increases; +, moderate increases; –, decreased level; **∞**, no change; ND, no data*.

## Conclusion

The molecular mechanisms by which *LRRK2, GBA, SNCA, PARK2, PARK6*, and *PARK7* defects trigger neuroinflammation and neurodegeneration in PD are poorly defined and need more studies. However, the abnormal function of *LRRK2, GBA, SNCA, PARK2, PARK6*, and *PARK7* genes has been linked to alteration in innate and adaptive immune responses in cancer, stroke, diabetes, male infertility, Crohn's disease, and infectious diseases (59, 96–98, 125, 127–133, 237–245, 290, 297, 298, 306–309). Findings from mouse models, cell system, and human specimens have shown that the abnormal expressions of *LRRK2, GBA, SNCA, PARK2, PARK6*, and *PARK7* genes and their corresponding proteins or enzymes (e.g., LRRK2, GCase, α-syn, parkin, PINK1, and DJ-1) are linked to the activation of MGCs, ACs, and NCs and the massive production of growth factors (e.g., GCSF, GMCSF, MCSF) and CCL and CXCL chemokines (i.e., CCL2/MCP1, CCL3/MIP1α, CCL4/MIP1β, CCL5/RANTES, CXCL1, and CXCL10), which are all accountable for the development and trafficking of immunological cells from the peripheral blood and bone marrow to the sites of inflammation for the generation of pro-inflammatory cytokines that lead to tissue destruction (61–69). The CCL2/MCP1, CCL3/MIP1α, CCL4/MIP1β, CCL5/RANTES, CXCL1, and CXCL10 chemokines are specific chemoattractants for tissue recruitment of several inflammatory subsets of MOs, Mφs, DCs, and CD4^+^ and CD8^+^ T cells (59, 60). Certain inflammatory conditions cause accelerated migration of immunological cell precursors out of the bone marrow and into the circulation (310–312). A similar condition is thought to occur in PD due to genetic defects in *LRRK2, GBA, SNCA, PARK2, PARK6*, and *PARK7* genes and the resultant alteration in the expression of their corresponding proteins or enzymes, i.e., LRRK2, GCase, α-Syn, parkin, PINK1, and DJ-1, which leads to the establishment of a network of several of the innate and adaptive immune cells, i.e., MOs and memory and effector T cells (43, 46–51). Hence, it is possible that immune cell integration and the resultant generation of pro-inflammatory cytokines at the periphery alter the blood–brain barrier integrity. This situation permits the recruitment of immune cells, to the specific region of the brain where infiltrated (e.g., MOs, DCs, CD4^+^ T cells, and CD8^+^ T cells) and residential immune cells (e.g., MGCs) meet and amplify their activation, and the resultant massive generation of pro-inflammatory cytokines (e.g., IFNγ, TNFα, IL-1β, IL-6, IL-8, IL-12, and IL-17), which are all lethal to DA neurons, and this condition develops neurodegeneration in PD.

## Author Contributions

AFM and SLH prepared and designed the tables. RR designed the figures and assisted in the writing and critical review of the text. MKP conceptualized, designed, wrote, reviewed, edited, and approved the submitted version of the manuscript. All authors contributed to the article and approved the submitted version.

## Conflict of Interest

The authors declare that the research was conducted in the absence of any commercial or financial relationships that could be construed as a potential conflict of interest. The handling editor declared a past co-authorship with the author MKP.
